# Association Between Non-Recovered Contrast-Associated Acute Kidney Injury and Poor Prognosis in Patients Undergoing Coronary Angiography

**DOI:** 10.3389/fcvm.2022.823829

**Published:** 2022-03-07

**Authors:** Dianhua Zhou, Zhubin Lun, Bo Wang, Jin Liu, Liwei Liu, Guanzhong Chen, Ming Ying, Huanqiang Li, Shiqun Chen, Ning Tan, Jiyan Chen, Yong Liu, Jianfeng Ye

**Affiliations:** ^1^Department of Cardiology, Dongguan TCM Hospital, Dongguan, China; ^2^Guangdong Provincial Key Laboratory of Coronary Heart Disease Prevention, Department of Cardiology, Guangdong Cardiovascular Institute, Guangdong Provincial People's Hospital, Guangdong Academy of Medical Sciences, Guangzhou, China; ^3^The First School of Clinical Medicine, Guangdong Medical University, Zhanjiang, China; ^4^Department of Cardiology, Zhongshan Hospital, Shanghai Institute of Cardiovascular Diseases, Fudan University, Shanghai, China

**Keywords:** recovered, non-recovered, contrast-associated acute kidney injury, coronary angiography, all-cause mortality

## Abstract

**Background:**

Previous studies have shown that renal function recovery after acute kidney injury (AKI) was associated with decreased risk of all-cause mortality. However, little is known about the correlation between renal function recovery and long-term prognosis in patients with contrast-associated acute kidney injury (CA-AKI) undergoing coronary angiography (CAG).

**Methods:**

We retrospectively enrolled 5,865 patients who underwent CAG. CA-AKI was defined as an increase in serum creatinine (SCr) ≥ 50% or ≥ 0.3 mg/dl from baseline within 72 h post procedure. Recovered CA-AKI was defined as a decrease in SCr to baseline or no CA-AKI level. The first endpoint was long-term all-cause mortality. Kaplan–Meier analysis and Cox regression analysis were used to investigate the association between kidney function recovery and long-term mortality.

**Results:**

During the median follow-up period of 5.25 years, the overall long-term mortality was 20.07%, and the long-term mortality in patients with recovered CA-AKI and non-recovered CA-AKI was 17.46 and 27.44%, respectively. After multivariate Cox hazard regression, non-recovered CA-AKI was significantly associated with long-term mortality, while recovered CA-AKI was not [recovered CA-AKI vs. no CA-AKI, hazard ratio (HR) = 1.06, 95% confidence interval (CI): 0.81–1.39, *p* = 0.661; non-recovered CA-AKI vs. no CA-AKI, HR = 1.39, 95% CI: 1.21–1.60, *p* < 0.001]. In the subgroup of CAD, both recovered CA-AKI and non-recovered CA-AKI were associated with increased risk of long-term all-cause mortality. However, in other subgroup analyses, only non-recovered CA-AKI was associated with increased risk of long-term all-cause mortality.

**Conclusion:**

Our results found that non-recovered CA-AKI is significantly associated with long-term mortality. In patients with CAD, recovered CA-AKI can still increase the risk of all-cause mortality. Clinicians need to pay more attention to patients suffering from CA-AKI, whose kidney function has not recovered. In addition, active prevention treatments should be taken by patients with CAD.

## Introduction

Contrast-associated acute kidney injury (CA-AKI) is a common complication after coronary angiography (CAG), with an incidence of 2–20% (1). It has been known in previous studies that CA-AKI involves serious clinical prognosis, such as increased rate of rehospitalization, end-stage renal failure, and mortality (2–6). The pathogenesis of CA-AKI is mainly related to hemodynamic changes in the kidney, the toxic effect, and oxidative stress of contrast agent on renal tubular cells (7, 8). Endothelin, nitric oxide, and prostaglandins, which are vasoactive substances, mediate changes in vascular motility, resulting in ischemic injury (9–11). A previous study demonstrated that among patients with acute kidney injury (AKI) during hospitalization, the long-term mortality of patients with renal function recovery was lower than that of patients without renal function recovery (12). In addition, patients with recovered renal function have a lower risk of short-term mortality in patients undergoing open abdominal aortic aneurysm surgery or with dialysis-requiring AKI (13, 14).

However, in the case of CA-AKI, few studies were conducted to explore the prognostic difference between recovery and non-recovery of renal function. Therefore, this study aims to assess the relationship between the recovery of renal function and long-term all-cause mortality in patients with CA-AKI, undergoing coronary angiography (CAG).

## Materials and Methods

### Enrollment and Treatment

**Figure 1:**
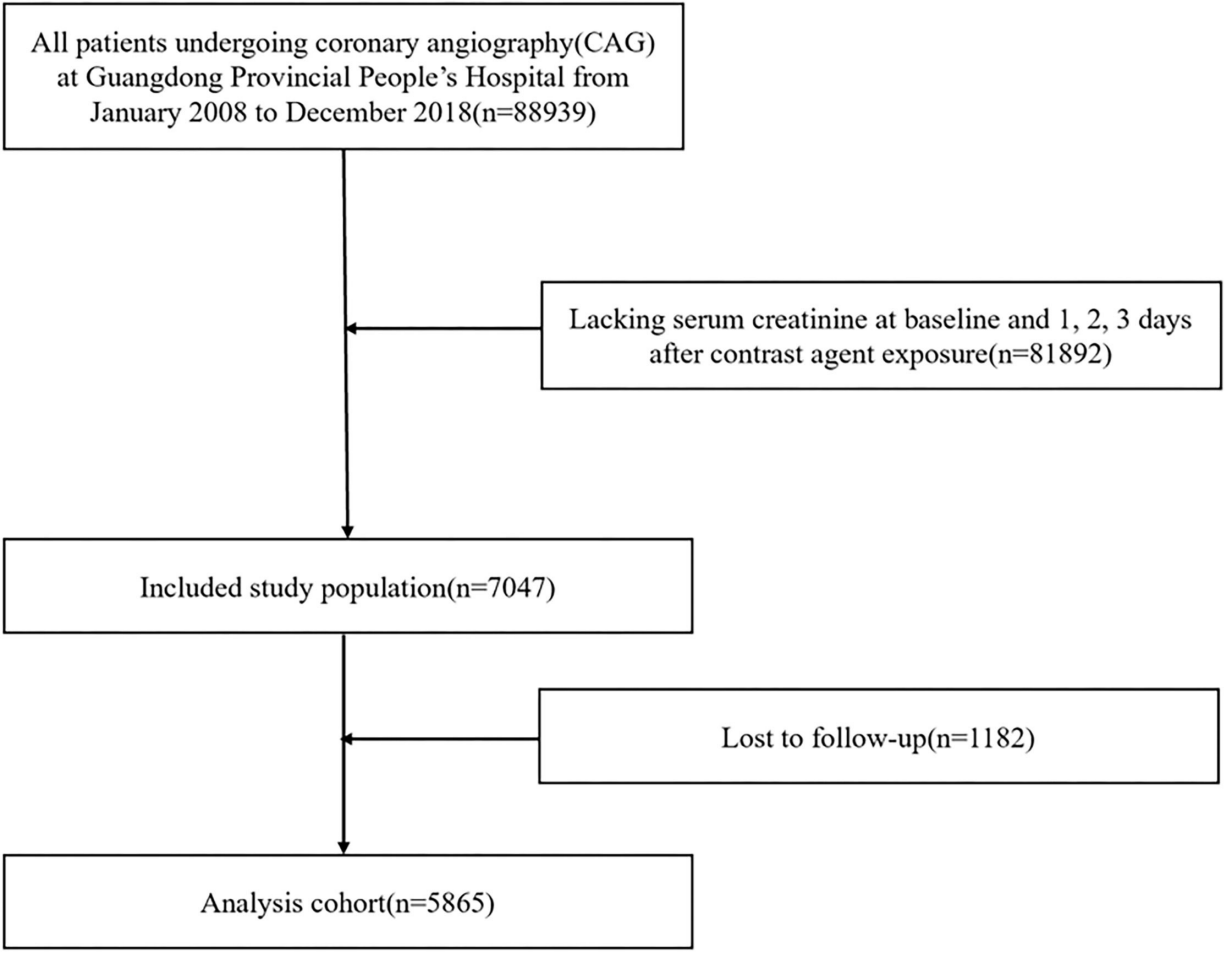
Study flow chart.

This observational study was conducted using data from the Cardiorenal ImprovemeNt (CIN)-I study, which was processed in the largest cardiovascular center in South China (Guangdong Provincial People's Hospital, China; ClinicalTrials.gov NCT04407936). The baseline information, including demographics, laboratory test results, mortality, and other clinical information, were extracted from the electronic clinical management records system of the Guangdong Provincial People's Hospital from January 2007 to December 2018. Follow-up data was monitored and recorded by trained nurses through outpatient interviews and telephonic follow-up. The follow-up information of patients lost to follow-up was retrieved from Guangdong Public Security System. Senior cardiologists were responsible for data quality control and periodical data verification. All patients undergoing CAG between January 1, 2007 and December 31, 2018 were screened. During this period, 88,938 patients had undergone CAG. We excluded patients (1) <18 years old (*n* = 0); (2) who lacked serum creatinine (SCr) concentration at baseline and 1, 2, 3 days after contrast agent exposure (*n* = 81,892); (3) with missing follow-up information on mortality (*n* = 1,182) (Figure 1). The study was approved by the Ethics Committee of Guangdong Provincial People's Hospital (No. GDREC2019555H) and conformed to the principles outlined in the Declaration of Helsinki. CAG and percutaneous coronary intervention (PCI) were executed by clinicians based on the patient's condition and the standard clinical practice guidelines.

### Endpoint and Definitions

The endpoint of our research was long-term all-cause mortality. CA-AKI was defined as an increase in SCr ≥ 50% or ≥ 0.3 mg/dl from baseline within 72 h after the CAG (15). Recovered CA-AKI was defined as a decrease in serum creatinine to baseline or no CA-AKI level according to the last measurement of SCr within 72 h after CAG. Non-recovered CA-AKI was considered when SCr was not recovered within 72 h. Coronary artery disease (CAD) was diagnosed by CAG and was depicted as 50% stenosis of at least one coronary artery. CAD was also determined according to the International Statistical Classification of Diseases and Related Health Problems 10th Revision (ICD-10). In addition, comorbidities included acute myocardial infarction (AMI), diabetes mellitus, hypertension, congestive heart failure (CHF), and chronic kidney disease (CKD). We used the Modification of Diet in Renal Disease (MDRD) equation to calculate the estimated glomerular filtration rate (eGFR), and CKD was defined as an eGFR <60 ml/min/1.73 m^2^ (16, 17). Anemia was defined as a baseline hematocrit value <39% for men or <36% for women according to the WHO criteria (18). CHF was defined as New York Heart Association (NYHA) functional class > 2, and Killip class > 1.

### Statistical Analysis

The study population was divided into three groups, namely no CA-AKI, recovered CA-AKI, and non-recovered CA-AKI. Continuous variables were compared using one-way analysis of variance (ANOVA), and categorical data were analyzed by the Pearson chi-squared test. The cumulative mortality was determined by Kaplan–Meier (K-M) analysis, and a log-rank test was used to assess differences between curves. Univariate and multivariate Cox regression analyses were used to evaluate the association between the two types of CA-AKI and long-term all-cause mortality. We performed a subgroup analysis of patients with CAD, CKD, hypertension, CHF, and diabetes mellitus. All data analyses were performed using R software (version 3.6.5; R Foundation for Statistical Computing, Vienna, Austria). A two-sided *p* < 0.05 indicated significance for all analyses.

## Results

### Clinical and Procedural Characteristics

A total of 5,865 consecutive patients who underwent CAG were enrolled in this study. All included patients were divided into three different groups: 4,401 (75.04%) patients with no CA-AKI, 338 (5.76%) patients with recovered CA-AKI, and 1,126 (19.20%) patients with non-recovered CA-AKI. The baseline clinical characteristics of all the patients are shown in Table 1. Overall, the mean age was 63.8 ± 10.7 years, with men accounting for 67.88% of the population. Patients diagnosed with CAD, diabetes mellitus, CHF, and CKD accounted for 67.03, 26.77, 22.11, and 37.03%, respectively.

**Table 1:** Baseline characteristics.

**Characteristic**	**Overall (*n* = 5,865)**	**No CA-AKI (*n* = 4,401)**	**Recovered CA-AKI (*n* = 338)**	**Non-recovered CA-AKI (*n* = 1,126)**	***P*-value**
Male, *n* (%)	3,981 (67.88)	3,062 (69.58)	214 (63.31)	705 (62.61)	<0.001
Age, year	63.82 (10.74)	63.72 (10.84)	63.39 (10.51)	64.37 (10.42)	0.146
Age>75 year, *n* (%)	926 (15.79)	694 (15.77)	47 (13.91)	185 (16.43)	0.535
CAD, *n* (%)	3,928 (67.03)	3,199 (72.77)	158 (46.75)	571 (50.71)	<0.001
AMI, *n* (%)	1,180 (20.20)	956 (21.83)	43 (12.76)	181 (16.07)	<0.001
Diabetes mellitus, *n* (%)	1,564 (26.77)	1,204 (27.49)	74 (21.96)	286 (25.40)	0.045
Hypertension, *n* (%)	2,966 (50.76)	2,327 (53.13)	145 (43.03)	494 (43.87)	<0.001
CKD, *n* (%)	2,172 (37.03)	1,581 (35.92)	102 (30.18)	489 (43.43)	<0.001
CHF, *n* (%)	1,292 (22.11)	849 (19.38)	81 (24.04)	362 (32.15)	<0.001
Anemia, *n* (%)	2,389 (41.00)	1,733 (39.70)	126 (37.28)	530 (47.15)	<0.001
WBC, 10^9^/L	8.31 (3.14)	8.37 (3.16)	8.01 (2.92)	8.20 (3.12)	0.055
RBC, 10^12^/L	4.40 (0.71)	4.42 (0.70)	4.46 (0.66)	4.31 (0.73)	<0.001
Albumin, g/L	34.89 (4.86)	34.91 (4.73)	35.45 (4.95)	34.66 (5.30)	0.032
MCHC, g/L	333.06 (12.36)	333.41 (12.21)	331.62 (12.87)	332.13 (12.68)	0.001
Hemoglobin, 10^12^/L	127.79 (19.79)	128.68 (19.21)	128.35 (20.01)	124.14 (21.44)	<0.001
Hematocrit	0.38 (0.06)	0.39 (0.06)	0.39 (0.06)	0.37 (0.06)	<0.001
Cys-C, mg/L	1.43 (0.82)	1.36 (0.74)	1.66 (1.37)	1.80 (0.95)	<0.001
eGFR, ml/min/1.73 m^2^	68.94 (28.86)	69.51 (28.21)	71.98 (29.10)	65.80 (31.03)	<0.001
ACEI/ARB, *n* (%)	1,979 (35.81)	1,698 (40.04)	79 (24.41)	202 (20.95)	<0.001
Beta-blocker, *n* (%)	3,717 (67.26)	2,998 (70.69)	187 (58.26)	532 (55.19)	<0.001
Statin, *n* (%)	3,772 (67.35)	3,160 (74.51)	139 (43.30)	423 (43.88)	<0.001
CMV, ml	138.22 (92.19)	144.32 (90.97)	126.75 (109.11)	117.79 (88.15)	<0.001
PCI, *n* (%)	3,110 (53.03)	2,603 (59.15)	119 (35.21)	388 (37.97)	<0.001

*CA-AKI, contrast-associated acute kidney injury; CAD, coronary artery disease; AMI, acute myocardial infarction; CKD, chronic kidney disease; CHF, chronic heart failure; WBC, white blood cell; RBC, red blood cell; MCHC, mean corpuscular hemoglobin concentration; eGFR, estimated glomerular filtration rate; ACEI/ARB, angiotensin-converting enzyme inhibitor/angiotensin receptor blocker; CMV, contrast media volume; PCI, percutaneous coronary intervention*.

The comorbidity rate of AMI, diabetes mellitus, PCI, and hypertension was higher in the normal group. Patients in the non-recovered CA-AKI group had lower contrast media volume as well as higher prevalence of CKD and CHF.

### Primary Outcomes

**Figure 2:**
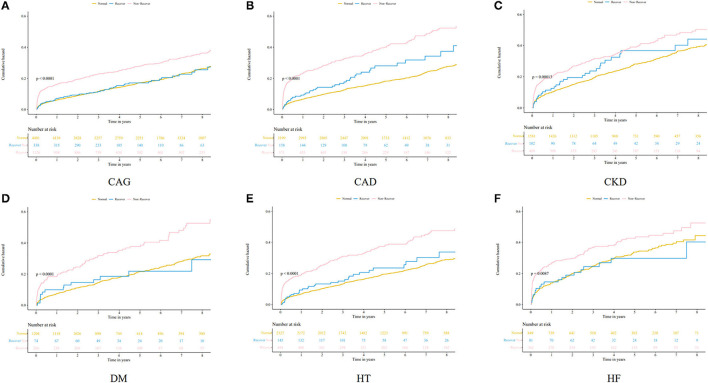
Kaplan–Meier curves for the cumulative probability of mortality stratified according to no contrast-associated acute kidney injury (CA-AKI), recovered CA-AKI, and non-recovered CA-AKI. **(A)** coronary angiography patients; **(B)** coronary artery disease patients; **(C)** chronic kidney disease patients; **(D)** diabetes mellitus patients; **(E)** hypertension patients; **(F)** chronic heart failure patients.

During the follow-up period (mean, 5.25 years), a total of 1,177 all-cause deaths occurred. The mortality in the three groups was 18.38% for no CA-AKI (809 patients), 17.46% for recovered CA-AKI (59 patients), and 27.44% for non-recovered CA-AKI (309 patients). There were significant differences in long-term mortality among patients without CA-AKI, patients with recovered CA-AKI, and patients with non-recovered CA-AKI in Kaplan–Meier curve analysis (*p* < 0.001) (Figure 2).

**Figure 3:**
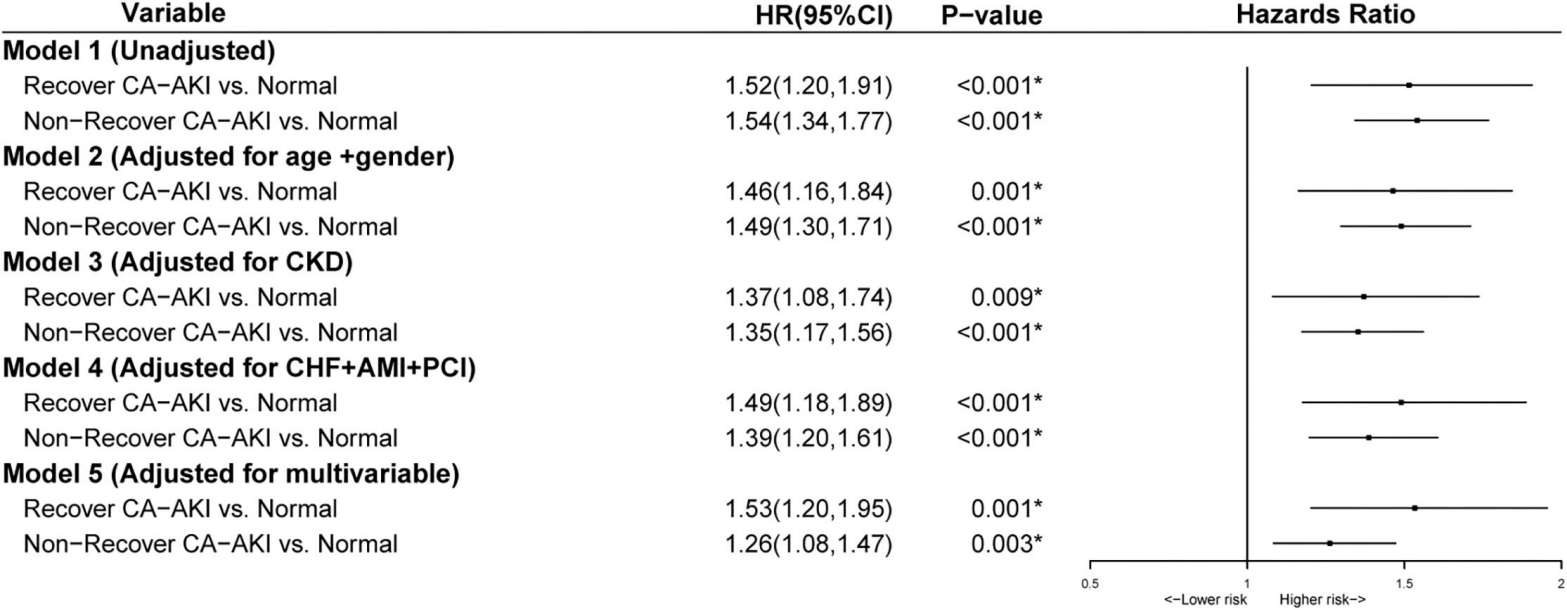
Multivariable analysis for mortality stratified according to no contrast-associated acute kidney injury (CA-AKI), recovered CA-AKI, and non-recovered CA-AKI. Model 1: Unadjusted; Model 2: Adjusted for age>75 years and gender; Model 3: Adjusted for chronic kidney disease (CKD); Model 4: Adjusted for chronic heart failure, acute myocardial infarction (AMI), and percutaneous coronary intervention (PCI); Model 5: Adjusted for age>75 years, gender, diabetes mellitus, anemia, chronic heart failure, hypertension, CKD, albumin, PCI, contrast media volume, and AMI. *p < 0.05.

After adjusting for age ≥75 years, sex, CKD, AMI, PCI, CHF, hypertension, diabetes mellitus, anemia, contrast media volume, and albumin, multivariate Cox regression analysis revealed that compared with no CA-AKI group, non-recovered CA-AKI group was significantly associated with increased mortality [adjusted hazard ratio (HR): 1.39, 95% confidence interval (CI):1.21–1.60, *p* < 0.001], while the recovered CA-AKI group was not (adjusted HR: 1.06, 95% CI: 0.81–1.39, *p* = 0.661) (Figure 3).

### Subgroup Analysis

**Figure 4:**
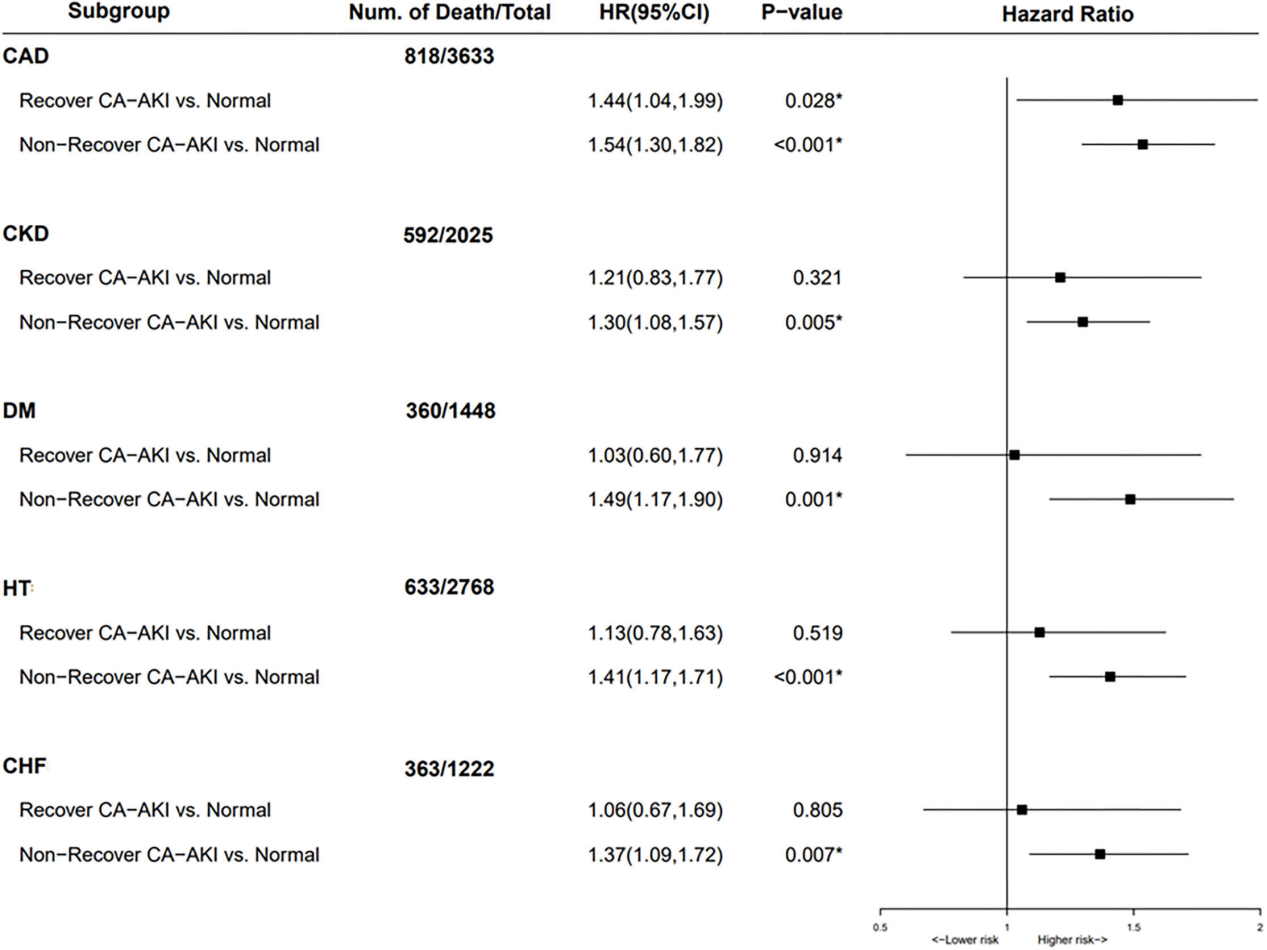
Subgroup analysis for mortality stratified according to no contrast-associated acute kidney injury (CA-AKI), recovered CA-AKI, and non-recovered CA-AKI. Adjusted for age>75 years, gender, diabetes mellitus, anemia, chronic heart failure, hypertension, chronic kidney disease (CKD), albumin, percutaneous coronary intervention (PCI), contrast media volume, and acute myocardial infarction (AMI). *p < 0.05.

In subgroup analysis for patients complicated with CAD, both patients with recovered CA-AKI and non-recovered CA-AKI had an increased risk of long-term mortality when compared to that of patients without CA-AKI (Figure 4). In the rest of the subgroup analysis, including CKD, CHF, hypertension, and diabetes mellitus, non-recovered CA-AKI group could still increase the risk of all-cause mortality compared with no CA-AKI group. However, there was no association between the recovered CA-AKI group and long-term all-cause death.

## Discussion

Our results showed that patients with non-recovered CA-AKI had a worse long-term prognosis, while the patients with recovered CA-AKI did not. We found similar results in various subgroups, such as CKD, diabetes mellitus, hypertension, and CHF groups. This study emphasized that clinicians need to pay more attention to patients with non-recovered CA-AKI in clinical practice.

According to SCr level before discharge, we divided CA-AKI patients into recovered CA-AKI groups and non-recovered CA-AKI groups. We found that the patients who recovered from CA-AKI were similar to patients without CA-AKI, based on long-term mortality. This was not consistent with what Wehbe et al. reported in their study (19). The reason could be that the lung transplant patients in the study by Wehbe et al. had more severe kidney immune damage. Similar results have been found in the study by Welten et al. (13). The reason could be that in comparison with our patients, patients who underwent aortic surgery in the study by Welten et al. had more blood loss, which could lead to severe AKI and increase the risk of long-term mortality. In addition, the study of Bhatraju et al. found that when compared with no AKI patients, recovered AKI patients and non-recovered AKI patients have a worse long-term prognosis. This may be related to the endpoint (20). The endpoint of the study by Pavan K. Bhatraju et al. is a major adverse kidney event, defined as incidence or progressive CKD, long-term dialysis, or all-cause death.

At present, most of the studies that focused on the factors and mechanism of renal function recovery are aimed at AKI patients. Hickson et al. and Schmitt et al. found that baseline eGFR and aging are important factors affecting the recovery of renal function (21, 22). This is consistent with our results. We know that when compared with recovered CA-AKI and no CA-AKI patients, non-recovered CA-AKI patients have a higher incidence of chronic heart failure (Table 1). This is similar to the results of Hickson et al., which showed that patients with heart failure are more difficult to recover from kidney damage. Furthermore, for patients with AKI, who require renal replacement therapy, maintaining kidney perfusion, avoiding hypotension, and avoiding nephrotoxins and technique-related infection can help in the recovery of the kidney (23). Future research needs to clarify the renal recovery mechanism of CA-AKI patients to reduce the burden of disease.

According to the subgroup analysis results, we confirmed that non-recovered CA-AKI was associated with an increased risk of long-term mortality in patients with complications, including CKD, diabetes mellitus, hypertension, and CHF. This further confirms our results and increases the generalizability of our results. Therefore, we should pay more attention to the prevention of non-recovered CA-AKI in the clinics to reduce its occurrence. At present, CA-AKI prevention mainly focuses on renal replacement therapy, drugs, and intravenous crystalloid (24). However, the benefits of preventive renal replacement therapy and most drugs have not been proven. Current guidelines recommend the use of isotonic saline to reduce the risk of CA-AKI (25). In a meta-analysis of 124 studies, it was found that compared with saline, statins, xanthine, N-acetylcysteine (NAC), and sodium bicarbonate could reduce the occurrence of CA-AKI (26). It was also necessary to reduce the dose of contrast medium for high-risk patients. Future research should pay more attention to clarifying the mechanism of CA-AKI and explore more preventive measures for CA-AKI.

### Limitations

Our research has several limitations. First, our study is a single-center retrospective analysis, which may lead to a lack of representativeness of our results. However, our study has a large sample size, and the results have been verified in multiple subgroups, which makes our results more credible. Second, long-term mortality is complex and multivariable. Due to the lack of other endpoint events, promotion of our results will be limited. Third, the definition of CA-AKI is diverse. In our research, CA-AKI was defined as an increase in SCr ≥ 50% or ≥ 0.3 mg/d from baseline within 72 h after the CAG. Fourth, we excluded patients who lacked SCr values at 72 h postoperatively, which resulted in the exclusion of 92% of the patients. Without the results of SCr at 72 h after surgery, we could not assess the recovery of renal function, and this in turn could lead to selection bias. However, we also performed a subgroup analysis, which makes our results more credible. Fifth, we were unable to obtain the patient's SCr during follow-up, which prevented us from assessing the long-term recovery of renal function after discharge. This has important implications for assessing whether a patient will develop CKD, as it has important implications for assessing long-term mortality.

## Conclusion

In conclusion, we found that non-recovered CA-AKI is associated with a worse prognosis. This means that we need to pay more attention to CA-AKI patients whose renal function has not recovered after CAG. Moreover, future research still needs to pay attention to the recovery mechanism of CA-AKI patients.

## Data Availability Statement

The raw data supporting the conclusions of this article will be made available by the authors, without undue reservation.

## Ethics Statement

The studies involving human participants were reviewed and approved by Ethics Committee of Guangdong Provincial People's Hospital (No. GDREC2019555H). The patients/participants provided their written informed consent to participate in this study.

## Author Contributions

DZ, ZL, BW, JL, NT, JC, YL, and JY contributed to the conception of the study. DZ, ZL, BW, JL, YL, JY, LL, GC, HL, MY, and SC contributed significantly to the data analysis and manuscript preparation. DZ, ZL, BW, JL, YL, and JY performed the data analyses and wrote the manuscript. DZ, ZL, BW, and JL contributed to the design and statistical analysis of this study. All authors have read and approved the manuscript.

## Funding

The study was supported by the National Natural Science Foundation of China (Grant nos. 81670339 and 81970311), the Beijing Lisheng Cardiovascular Pilot Foundation (Grant no. LHJJ201612127), the Science and Technology Planning Project of Guangdong Province (Grant no. 2014B070706010), the Science and Technology Planning Project of Guangzhou (Grant no. 201704020124), Dengfeng Project in Guangdong Province (DFJH201919 and DFJH2020026), and Dongguan Social Science and Technology Development Key Project (202050715002176).

## Conflict of Interest

The authors declare that the research was conducted in the absence of any commercial or financial relationships that could be construed as a potential conflict of interest.

## Publisher's Note

All claims expressed in this article are solely those of the authors and do not necessarily represent those of their affiliated organizations, or those of the publisher, the editors and the reviewers. Any product that may be evaluated in this article, or claim that may be made by its manufacturer, is not guaranteed or endorsed by the publisher.
